# Integrating Social Presence With Social Learning to Promote Purchase Intention: Based on Social Cognitive Theory

**DOI:** 10.3389/fpsyg.2021.810181

**Published:** 2022-01-20

**Authors:** Miao Li, Ying Hua

**Affiliations:** School of Information Technology and Management, University of International Business and Economics, Beijing, China

**Keywords:** live streaming commerce, social presence, social learning, vicarious learning, purchase intention, social cognitive theory

## Abstract

Consumers mainly acquire information through social learning in online shopping environment, and social presence as a media attribute generated by real-time interactions in live streaming commerce is more conducive for consumers’ social learning. Therefore, it is worth investigating the roles of social presence and social learning on consumers’ purchase intention (PI) in the strong interactive environment. Based on social cognitive theory (SCT) framework and drawing on social presence theory and social learning theory, this study investigates the relationships among social presence, social learning process, and PI. Social presence is operationalized into social presence of others (SPO) and social presence of interactions (SPI), whereas social learning process contains external interaction process [exploitative learning (ETL) and exploratory learning (ERL)] and internal psychological process [cognitive appraisal (CAP) and affective appraisal AAP)]. The results from a survey of 372 consumers of live streaming commerce indicate that SPO and SPI positively affect ETL and ERL and then contribute significantly to the building of CAP and AAP, which can lead to PI. The findings also provide guidance for brand managers and retailers in building more effective interactive atmosphere and promoting consumers’ positive attitude toward brands in live streaming marketing.

## Introduction

The live streaming industry has been booming in recent years and is even more common and popular in China, especially during the coronavirus pandemic. Up to December 2020, the number of live streaming commerce users in China is 388 million (CNNIC, 2021). As a typical representative of digital economy, live streaming commerce reshapes consumers’ consuming patterns and decision-making process. Marketers are gradually aware of the unique features of live streaming commerce, such as live stream and real-time interactions, and have adopted live streaming marketing to attract consumers’ attention and promote their purchase intention (PI). For example, by the end of 2020, more than 90% of brands have launched live streaming marketing on Tmall, according to the 2020 Taobao Live Streaming Ecological Development Report (Taobangdan, 2020). Due to the fierce competition, high cost and low premium ability in live streaming marketing strategy cooperating with internet celebrities, an increasing number of brands start “brand live streaming” to enhance brand autonomy and long-term development. However, compared with internet celebrity live streaming, brand live streaming lacks fans’ advantage and user stickiness, resulting in a large gap in sales transformation with the former. In this case, how can brands consider other aspects to promote the sales transformation in brand live streaming? This is one of the vital problems in the operation and development of brand live streaming marketing.

Recently, the drastically growing live streaming commerce practice has also attracted the attention of academic society. The existing research related to consumers’ behavior mainly focuses on platform technology, broadcaster’s characteristics, perceived value, etc. (Gefen and Straub, 2004; Ang et al., 2018; Osei-Frimpong and Mclean, 2018). Compared with traditional e-commerce, the characteristics of real-time video and real-time interactions in live streaming commerce bring a high degree of social presence. In internet marketing research, scholars believe that social presence, as a media attribute, is a critical factor for consumers’ purchase decision. However, social presence created by three-dimensional and real-time interactions in live streaming commerce has been ignored. It is worthwhile to learn more about the mechanism of consumers’ behaviors in live streaming commerce, which is significant for brand sustainability in the cruel market competition.

In online shopping environment, consumers mainly obtain information about products or services through social learning, especially vicarious learning. Consumers that acquire more knowledge about brand or products will have a more positive attitude toward the brand, therefore promoting brand loyalty and brand trust and affecting PI afterward (Zhao et al., 2019). Myers (2018) proposed the concept of coactive vicarious learning (CVL), which refers to “individuals learn others’ experience and knowledge in communication and interaction.” Live streaming commerce provides a more convenient, more efficient, and more real learning platform for consumers (Li et al., 2020). For instance, the particular affordances of live streaming commerce enable consumers to share experience or consult problems with others, especially with the sellers (Chen and Lin, 2018), which is consistent with CVL. Therefore, consumers’ CVL in live streaming commerce provides a new perspective to explore the influence mechanism of social presence on PI.

Furthermore, social presence has a positive effect on online learning performance and satisfaction in e-learning situation (Richardson et al., 2017), and consumers’ social learning in network environment can also be regarded as online learning. However, the black box between social presence which enables vicarious learning and the subsequent psychology and behavior in live streaming commerce also remains unclear. Prior studies highly emphasize the significance of social presence in distance learning and the importance of CVL in interactive environment as well. However, many points are also unclear, such as the dimensions of CVL, the impact of social presence on CVL, and the subsequent effects on consumers’ psychology and behavior.

To address these problems, this work tries to explore three research questions of (1) How does social presence affect CVL? (2) What internal psychological process would be influenced by CVL? (3) How internal psychological process affect PI?

Using social cognitive theory and social learning process framework, we build a model to explain the influential mechanism of social presence on PI, considering the mediating role of CVL and internal psychological process. The paper proceeds as follows. The first section provides theoretical background, including discussing social presence, social learning theory, and CVL. Next, we construct the research model and the hypotheses. In the subsequent sections, the paper presents the research methodology and study results. Finally, we discuss the research findings and implications, and also the limitations and future research prospects.

## Literature Review and Theoretical Background

### Social Cognitive Theory

Social cognitive theory (SCT) was first proposed by Bandura (1986). Then, scholars widely applied the ideas and methods of this theory and carried out a large number of empirical studies. Traditional behavioral theory points out that individual behavior depends entirely on external environmental stimuli. However, this view ignores the interrelationship between environmental stimuli and behavior. Bandura believes that the emergence of individual behavior does not completely depend on the external environment and puts forward SCT. This theory analyzes the influencing factors of individual behavior in detail and holds that the generation or change of individual behavior is not only affected by external environmental factors, but also influenced by their own internal psychological factors (Bandura, 1986).

Social cognitive theory is widely used by researchers to analyze the influencing factors of individual behavior (Sumak et al., 2011; Chiu and Tsai, 2014; Zhou et al., 2020). Thus, SCT also provides a theoretical basis for this paper to study the factors for consumers’ PI. As a media attribute of the live streaming commerce, social presence is the external environment that affects individuals’ social learning process and then affects their behavior intention.

### Social Presence Theory

Social presence was initially conceptualized by Short et al. (1976) and was defined as the salience of other members and the resulting salience of interpersonal interactions in a mediated conversation. Walther (1992) and Rice (1993) continued this definition in their studies afterward. Biocca et al. (2003) also emphasize that social presence refers to the degree to which an individual feels access to other people’s intentions and sensory impressions through mediated communication. The majority of definitions of social presence indicated that social presence is the psychological evaluation of other people’s real presence by media users (Shen, 2012; Lee and Shin, 2014) and interpersonal connection (Lowenthal, 2010).

Social presence is considered as an attribute of a communication medium and closely related to intimacy and psychological closeness (Short et al., 1976). In previous e-commerce studies, social presence has been adopted as a unidimensional concept which is often measured by perceived warmth, sociability and sensitivity (Rice and Case, 1983; Gefen et al., 2003). Shen et al. (2010) subdivided the construct of social presence into three dimensions of awareness, cognitive social presence, and affective social presence. In online learning environment, Sung and Mayer (2012) explored five factors representing facets of social presence, namely social respect, social sharing, open mind, social identity, and intimacy. However, these dimensions mostly focus on human warmth conveyed by the computer-mediated medium or interpersonal connections, without distinguishing the salience of others’ presence and interpersonal interactions. Tu (2002) proposes three-dimensional model of social presence in online learning communities, namely social context, online communication, and interactivity. Furthermore, Lu et al. (2016) propose a three-dimensional model of social presence in social commerce context, namely social presence of web, social presence of others (SPO), and social presence of interactions (SPI), respectively. Live streaming commerce is one form of social commerce; thus, social presence in live streaming commerce should also be a multidimensional construct.

The real-time interactive characteristic in live streaming commerce can bring consumers a high degree of social presence with regarding as a media attribute (Tafesse, 2016). Differentiating with online learning conditions, consumers in live streaming environments mainly interact with streamer and other consumers, rather than the computer-mediated medium. Therefore, our conceptualization of social presence is akin to the model of Lu et al. (2016), but only considering two dimensions of SPO and SPI. SPO, also called awareness, means the extent to which other members appear to exist or respond to members in a virtual community (Shen et al., 2010). In live streaming commerce, various social cues generated by consumers such as textual information on bullet screen including reviews or referrals, and also prompt message thrown by the system such as the number of viewers, followers, likes, and purchasing behavior and information, will increase perception of other online consumers and their actions. Another dimension is SPI. Both Tu (2002) and Caspi and Blau (2008) emphasized that interactivity is one important dimension of social presence. In live streaming commerce, computer-mediated communication forms (e.g., real-time video and bullet screen) have been employed as efficient means for interactions between sellers and customers.

### Social Learning Theory

Bandura and McClelland (1977) proposed social learning theory, which emphasizes social factor. Social learning is essential learning information by observing the behaviors of other members (Bandura and McClelland, 1977; Lorenzo et al., 2012). In social networking environments, one important social psychology is social learning, with learning from the experience and actions from others. Consumers can not only observe the information displayed by other customers, such as reviews and recommendations but also have access to social experiences and actions by communicating with streamers and other customers in live streaming commerce (Li et al., 2020). Individuals would become clear about whether the products meet their demand, what are worth buying, and how is the shopping experience. The learning behavior may positively affect customers’ attitude and influence their PIs afterward (Lorenzo et al., 2012).

Social learning theory underlines that social learning should first obtain new information and behavior patterns through observation and then internalize the observed information, which can be regarded as a process of internal processing of the external information. From this standpoint, social learning process involves two processes: an external interaction process and an internal psychological process (Illeris, 2003). The external interaction process is a social level, such as observation, participation, communication, and cooperation, which promotes the integration of social environment and individual. The internal psychological process is two equal psychological functions, including the cognitive function dealing with the learning information, and the emotional function representing psychological response (Illeris, 2003). Accordingly, the internal psychological process can be separated into two dimensions, namely cognitive dimension and affective dimension.

In our research, the external interaction process involves consumers’ interaction with streamers and other customers, namely CVL, whereas the internal psychological process involves cognitive response and emotional response after interacting with others, introducing cognitive appraisal (CAP) and affective appraisal (AAP), respectively. CAP refers to the utilitarian aspect of attitude which is an evaluation based on beliefs and knowledge structures, whereas AAP is an estimation based on emotions, feelings, and reactions (Lee et al., 2012).

### Coactive Vicarious Learning

Since learning from experience of other people has long been regarded as an essential factor for success, Gioia and Manz (1985) proposed the concept of vicarious learning and emphasized that vicarious learning means an individual learns from others’ experience and actions, rather than from his/her own behavior or experience. Furthermore, Myers (2018) proposed the concept of CVL, which emphasizes on a learning process in which learners and others consciously share and participate together and learn others’ experience through interpersonal interactions, so as to jointly construct a situated understanding of objects. CVL includes three elements of experience, analysis, and support (Myers, 2018). Experience refers to the “raw material,” providing the essential information for engaging in a CVL interaction that contains both interpersonal sharing and reflecting on multiple experiences. In addition, analysis means individuals evaluate and investigate the experience obtained in interactions to form new understanding, taking the forms of questions, judgments, or requests for elaboration. The third component of CVL is support, namely an individual’s social support or emotional support in the interpersonal interactions. Compared with traditional e-commerce, live streaming commerce provides more opportunities for CVL, owing to its distinctive affordances (Chen and Lin, 2018; Sun et al., 2019). When a streamer shows products, it is available for consumers to communicate with the streamer and other customers in real time by giving comments for further analysis and comparison.

Although Myers (2018) has put forward the concept of CVL and its components, the dimensions of this construct are not clarified. In organizational learning context, Brady and Davies (2004) showed a model of project capability-building consisting of exploratory learning (ERL) and exploitative learning (ETL) which are related to unfamiliar activities and routine activities, respectively. Drawing upon Brady and Davies (2004), this study attempts to divide CVL into two dimensions of ETL and ERL, from the perspective of ambidextrous learning (Gibson and Biekinshaw, 2004). ETL is characterized by refining, screening, selecting, etc., whereas ERL is characterized by searching, attempting, discovering, and innovating, etc. On the one hand, consumers can learn information by refining or choosing the content of the products that currently showed and experienced by sellers through interactions, which is consistent with ETL. On the other hand, customers are also able to gain information by attempting or discovering new knowledge of products or skills that meet their need or preference through interactions, which is in accordance with ERL.

## Research Model and Hypotheses

### Social Presence and Coactive Vicarious Learning

When shopping online, consumers are able to perceive the existence of other customers based on a variety of social cues (Cialdini, 2001), such as online review, product recommendations, and transactional history. The perception of SPO helps consumers to learn the experience of other members (Dunlap and Lowenthal, 2009), and consumers’ attitudes and behaviors tend to be affected by persuasion from similar individuals (Cialdini, 2001). Particularly, in live streaming commerce, buyers can easily learn interactive and behavioral information of other buyers, such as others’ comments, consultations, purchasing behaviors, and so on. If social cues release positive signals, consumers will have more confidence in the seller’s ability and integrity, and their trust toward the seller will also increase. According to Mardsen (2010), consumers can learn from and influenced by the experiences and behaviors of other people who they trust.

In social commerce environments, Lu et al. (2016) suggest that perception of others has a positive impact on trust in online sellers. Meanwhile, customers tend to be promoted to participate and interact with others once they perceive high trustworthiness with others (Wongkitrungrueng and Assarut, 2020). When an individual has doubts or confusion about product introduced by seller, he/she would be more willing to interact with the seller, thus further and clearly understanding other aspects of the product, such as maintenance knowledge, using skills, and purchase process. Besides, if consumers prefer to search or discover the information of new products meeting their needs or preferences, they could also communicate with the seller to obtain new related knowledge. Therefore, we have the following hypotheses:

**H1a.** SPO positively affects ETL.
**H1b.** SPO positively affects ERL.

Meanwhile, social presence theory emphasizes that the high interactivity of medium can produce high social presence and enhance the perception of interaction with others (Fulk et al., 1987). Live streaming commerce is based on the interactions between consumers and others (especially the sellers) to learn more real experience and evaluation of products and brands. These interactions can be regarded as parasocial interaction, which reflects the mutual interaction perceived by both sides (Hartmann and Goldhoorn, 2011). In online learning condition, text-based online discussion, as an interactive media tool, will positively affect online learning (Caspi and Blau, 2008). The chat tools in live streaming commerce convey a sense of communication. Consumers can also understand the attitude and integrity of sellers through these tools, thus building trust, which is conducive for consumers to learn more relevant information from the seller. Social presence plays a significant role in social interaction and is also a factor to promote members’ engagement behavior (Shen et al., 2010). If the seller replies quickly and targeted to buyers’ consultations in product introduction in live streaming commerce, consumers would be more satisfied with and trust in the seller, which will contribute to interactions between consumers themselves and the seller. Meanwhile, the timely and effective response from sellers to consumers’ questions will make consumers perceive a high degree of interactivity. Furthermore, perceived interactivity conveys a high sense of social presence and then promotes interactions such as information exchange and two-way response (Song and Zinkhan, 2008).

As previously mentioned, consumers could get more accurate knowledge about products presented by sellers or explore new information in accord with their own requirements through interacting with sellers in live streaming commerce. This discussion informs the following hypotheses:

**H2a.** SPI positively affects ETL.
**H2b.** SPI positively affects ERL.

### Coactive Vicarious Learning and Internal Psychological Process

Social interaction is the basis of cognitive development (Lee and Kozar, 2009). Previous studies have shown that social interactions with others often affect their own beliefs, attitudes, and behaviors (Godes et al., 2005). In live streaming commerce, brand sellers help consumers to form a preliminary understanding of the product experience that is more real than traditional e-commerce through interactions, thus promoting positive attitude toward the seller (Chen and Lin, 2018). Researchers stressed that social commerce is not only limited to the dissemination and sharing of product information, but also provides a way to continuously interact with sellers and share experience to deepen the relationship between them (Ashley and Tuten, 2015). Therefore, as a special form of social commerce, live streaming commerce bridges the gap between consumers and sellers.

Live streaming commerce enables consumers to interact with sellers in real time to acquire more detailed product knowledge. It allows consumers to analyze the experience of the product currently introduced by consulting or commenting, and to further compare with the information obtained previously, and then to interact with sellers again or more. In this in-depth interaction process, consumers could gain more accurate information to form their own understanding of product experience. In addition, if the product introduced by the seller does not meet consumers’ needs or preferences, they can also interact with the seller directly and propose their targeted demands for products, such as product basic parameters, product efficacy, and personal budget. After receiving consumers’ requirements, the seller shall directly recommend products or assist them to gather new information they need. Such interaction process is more targeted, so that consumers obtain new knowledge of the products they prefer more efficiently, comprehensively and timely.

Overall, sellers’ interactions provide utilitarian knowledge and help consumers with their cognition of the purchasing decision. Additionally, Illeris (2003) suggested that the external interaction process affects the internal psychological process of information acquisition and refinement. Therefore, we have the following hypotheses:

**H3a.** ETL positively affects CAP.
**H3b.** ERL positively affects CAP.

In the meantime, sharing experiences with others can have a positive impact and commitment in the relationship and develop emotional ties by supporting (Myers, 2018). Sellers can provide corresponding support to consumers through rewards or activities, and consumers can also give the seller support like clicking “attention” or “likes.” Social support is important for both sides to establish trust and to promote consumers’ emotional commitment toward the seller (Chen and Shen, 2015). In live streaming commerce, if the products introduced by the seller match their own needs, consumers will conduct in-depth interactions for the products introduced. Consumers can receive emotional support in interpersonal interactions, which will bring them warmth and satisfaction, and further enhance their positive emotion toward the seller. If the products introduced by the seller are not consistent with their demands, they can directly state their own needs or preferences. This kind of interaction can exactly meet the needs of consumers, thus providing better consumption experience and making them feel more valuable. Furthermore, the better experience of the interaction process, the more satisfied for consumers with sellers, thus generating higher emotional attachment (Chen A. H. et al., 2017). Therefore, we make the following presumptions:

**H4a.** ETL positively affects AAP.
**H4b.** ERL positively affects AAP.

### Internal Psychological Process and Purchase Intention

According to ABC attitude theory, cognition and affect are two dimensions of attitude (Illeris, 2003). Attitude plays an indispensable role in customers’ purchase decision. Researchers believe that there is direct relationship among consumers’ cognition, affect, and behavioral intention (Lee and Chen, 2011). Specifically, both technology acceptance model (TAM) and theory of planned behavior (TPB) emphasize that cognition and affect are two important predictors of consumer behavior. Consumers always evaluate the utilitarian value and hedonic value, and such appraisals can make their purchase decisions more reasonably (Chen et al., 2013). When the perceived utilitarian value and perceived hedonic value are high, consumers are more willingly to purchase from this seller rather than other sellers (Wang et al., 2013). Through appraising social information and affective experiences, consumers would have better understanding of product quality, seller quality, and also the level of experience satisfaction, so as to increase trust toward the seller. Further, trust in seller has positive effect on brand trust and affects consumer’s PI (Zhao et al., 2019). Based on the above discussion, we propose the following hypotheses:

**H5a.** CAP positively affects PI.
**H5b.** AAP positively affects PI.

**FIGURE 1:**
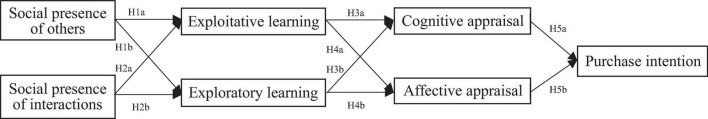
Research model.

The research model proposed in this study is illustrated in Figure 1.

## Methodology

### Samples and Procedures

In this paper, we used two samples to empirically test the theoretical model. Initially, 30 graduate students with engagement experience of brand live streaming commerce participated in the pilot test. Prior to the survey, we invited one marketing processor and three Ph.D. students to check the wording, legibility, and applicability and to validate the questionnaire to assure its accuracy and effectiveness. The results indicated that the structure and content of the questionnaire were available to conduct a large-scale empirical examination. Next, the formal investigation was carried out by Sojump.com which is a professional company providing questionnaire services in China. To ensure that all the participates were conformed to the research target, the respondents were asked to recall a recent or most impressive shopping experience of brand live streaming in Taobao and to complete the questionnaire according to this experience. We also controlled that only one response could be submitted in each IP address. In total, 372 available responses were collected, after deleting invalid and incomplete answers.

Among the participants we surveyed, about seventy percent of consumers are women (*n* = 261; 70.2%) and the rest (*n* = 111; 29.8%) are men. In terms of shopping experience of live streaming commerce, approximately 97.6% of the participants indicated that they shopped in live streaming commerce one or more times within half a year. About 90% of respondents are aged between 20 and 35 years old, and the education level of respondents is relatively high, with 51.6% from college and 44.6% from graduate school. The respondents’ demographic information is summarized in Table 1.

**TABLE 1:** Descriptive statistics of respondents.

Category	Item	Frequency	Percentage
**Gender**			
	Male	111	29.8%
	Female	261	70.2%
**Shopping frequency**			
	1 or more times in a month	260	69.9%
	1 time in a quarter	88	23.7%
	1 time in half a year	15	4.0%
	1 time in a year	6	1.6%
	Never	3	0.8%
**Age**			
	<20	4	1.1%
	20–25	122	32.8%
	26–30	103	27.7%
	31–35	110	29.6%
	>35	33	8.9%
**Education**			
	High school and below	14	3.8%
	College	192	51.6%
	Graduate school	166	44.6%

### Questionnaire and Measures

Questionnaires were developed to estimate the relationships among social presence, CVL, CAP, AAP, and PI. The items were adapted from the previous studies and modified properly to fit this research context. The measurement scales of SPO and SPI were derived from Gefen and Straub (2004) and also those designed by Lu et al. (2016), with being slightly revised in light of live streaming commerce environment. We developed the items of ETL and ERL based on the scales of exploitation and exploration from Zhou and Wu (2010) for this study. The scales of CAP and AAP were measured using four and five questions separately, adapted from Lee and Chen (2011) and Lee et al. (2012). We revised the three items from Prentice and Loureiro (2018) to assess PI. The measurement items are shown in Table A1. All these measures followed a seven-point Likert scales from 1 (not agree at all) to 7 (absolutely agree). As the data were collected in China, and to ensure that the meanings of questions were precisely captured, a back-translation process was conducted following the method used by Lu et al. (2016). Meanwhile, following the pilot test, the wordings of some items were slightly modified in Chinese questionnaire.

## Data Analysis and Results

We adopted the partial least squares (PLS) method and used Smart-PLS software to evaluate the measurement model and structural model. Since PLS method has minimal demands on measurement scales, sample size, and also model complexity, so we made these selections. Based on two-step data analysis process, we first conducted the assessment of the measurement model with the evaluation of reliability, convergent validity, and discriminant validity and then tested the structural model to evaluate the hypotheses (Henseler et al., 2009).

### Measurement Model

We evaluated the reliability of the constructs with Cronbach’s alpha (α) and composite reliability (CR). As shown in Table 2, the Cronbach’s alpha (α) for all concepts ranged from 0.709 to 0.774, and the composite reliabilities were all above 0.7, which means a favorable reliability (Chin, 1998). In addition, we evaluated convergent validity using the factor loadings and the average variance extraction (AVE). As shown in Table 2, all factor loadings were more than 0.6, and all AVEs were above 0.5, indicating satisfactory convergence validity (Chin, 1998). Finally, we evaluated discriminant validity using two methods. First, according to Fornell and Larcker (1981), we contrasted the square roots of the AVEs with construct correlations. Results showed that all the square roots of the AVEs were greater than the corresponding construct correlations (Table 3). Second, we also examined the heterotrait-monotrait ratio (HTMT) (Henseler et al., 2015). According to Table 3, the values of HTMT were all lower than 0.85. Thus, it was concluded that the discriminant validity was adequate.

**TABLE 2:** The assessment of measurement model for constructs.

Constructs	Items	Mean	S.D.	VIF	Loading	α	CR	AVE
Social presence of others	SPO1	6.10	0.884	1.178	0.721	0.724	0.800	0.572
	SPO2	5.91	0.887	1.279	0.771			
	SPO3	6.14	0.903	1.265	0.775			
Social presence of interactions	SPI1	5.73	1.037	1.431	0.739	0.730	0.832	0.552
	SPI2	5.86	1.013	1.365	0.734			
	SPI3	5.72	1.117	1.453	0.767			
	SPI4	5.73	1.082	1.300	0.733			
Exploitative learning	ETL1	5.94	1.070	1.406	0.731	0.712	0.822	0.536
	ETL2	5.80	1.086	1.284	0.724			
	ETL3	5.64	1.118	1.301	0.731			
	ETL4	5.79	1.135	1.380	0.752			
Exploratory learning	ERL1	5.87	1.151	1.435	0.758	0.731	0.832	0.553
	ERL2	5.87	1.081	1.380	0.738			
	ERL3	6.72	1.156	1.315	0.723			
	ERL4	6.07	0.955	1.386	0.755			
Cognitive appraisal	CAP1	5.74	1.076	1.519	0.820	0.774	0.869	0.688
	CAP2	5.44	1.325	1.611	0.839			
	CAP4	5.68	1.097	1.657	0.830			
Affective appraisal	AAP1	6.01	0.933	1.286	0.707	0.709	0.821	0.534
	AAP2	5.78	0.973	1.366	0.718			
	AAP4	5.80	1.101	1.418	0.781			
	AAP5	6.00	0.886	1.278	0.715			
Purchase intention	PI1	6.04	0.900	1.236	0.707	0.723	0.828	0.546
	PI2	5.89	0.954	1.422	0.763			
	PI3	5.95	0.950	1.401	0.740			

*α, Cronbach’s alpha; CR, composite reliability; AVE, average variance extracted; VIF, collinearity statistics. Some items were deleted because of the lower factor loadings.*

**TABLE 3:** Discriminant validity analysis.

Constructs	SPO	SPI	ETL	ERL	CAP	AAP	PI
SPO	0.756						
SPI	0.514	0.743					
ETL	0.496	0.629	0.732				
ERL	0.469	0.657	0.641	0.744			
CAP	0.399	0.512	0.589	0.569	0.830		
AAP	0.524	0.610	0.581	0.559	0.634	0.731	
PI	0.459	0.580	0.482	0.553	0.597	0.726	0.739
**Heterotrait-monotrait ratio (HTMT)**							
SPO							
SPI	0.794						
ETL	0.740	0.713					
ERL	0.723	0.559	0.818				
CAP	0.575	0.672	0.786	0.778			
AAP	0.786	0.839	0.809	0.785	0.765		
PI	0.681	0.794	0.667	0.777	0.796	0.825	

*SPO, social presence of others; SPI, social presence of interactions; ETL, exploitative learning; ERL, exploratory learning; CAP, cognitive appraisal; AAP, affective appraisal; PI, purchase intention.*

In addition, as shown in Table 2, the variance inflation factors (VIFs) for all the variables were below 4.00, and thus, it did not exist a serious multicollinearity problem (Hair et al., 2009). Common methods variance (CMV) is one of the sources of the measurement error caused by the characteristics of data sources (Luo et al., 2010). The extent of CMV was estimated in two tests. We first used Harman’s one-factor test by including all items with principal component factoring. The variance explained by the largest factor was 34.995%, lower than the critical value of 50%. Second, the correlation matrix among latent variables (Table 3) showed that the correlation coefficients among the variables were less than 0.9, indicating that CMV was not serious (Pavlou et al., 2007). Thus, we concluded that the CMV was improbably to misrepresent the results.

### Structural Model and Hypotheses Testing

**FIGURE 2:**
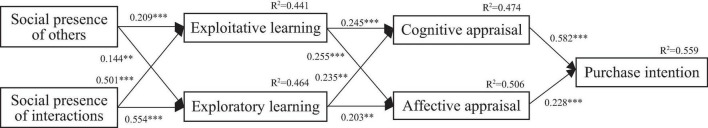
Structural model testing results. ^**^Significant level 0.01; ^***^Significant level 0.001.

As shown in Figure 2, the coefficients of determination (*R*^2^-values) were 0.441 for ETL, 0.464 for ERL, 0.474 for CAP, 0.506 for AAP, and 0.559 for PI, indicating an acceptable level of predictive power. In this study, the standardized root mean square residual (SRMR) of the structural model was 0.065, which is less than the 0.08 proposed by Hair et al. (2014). Besides, we evaluated the model’s predictive relevance using Stone-Geisser’s *Q*^2^-value as well. The *Q*^2^-values of ETL, ERL, CAP, AAP, and PI were all above 0, with 0.226, 0.221, 0.314, 0.255, and 0.299, respectively, suggesting that the model has predictive relevance (Hair et al., 2014). All these results demonstrate a satisfactory level of explanatory power and a good model fit.

According to the results shown in Figure 2 and Table 4, several findings were obtained. Specifically, SPO was found to have positive influences on both ETL (β = 0.209; *p* < 0.001) and ERL (β = 0.144; *p* < 0.01), in support of H1a and H1b. SPI was also found to be positively related to ETL (β = 0.501; *p* < 0.001) and ERL (β = 0.554; *p* < 0.001), thus supporting H2a and H2b. In addition, ETL (β = 0.245; *p* < 0.001) and ERL (β = 0.235; *p* < 0.01) significantly affected CAP, and thus, H3a and H3b were supported. Meanwhile, both ETL (β = 0.255; *p* < 0.001) and ERL (β = 0.203; *p* < 0.01) were also positively associated with AAP, supporting H4a and H4b. Moreover, CAP (β = 0.582; *p* < 0.001) and AAP (β = 0.228; *p* < 0.001) had significant influences on PI, and thus, H5a and H5b were supported.

**TABLE 4:** Results of path analysis.

Paths	Path coefficient	*t*-value	Support
SPO → ETL	0.209***	3.585	H1a supported
SPO → ERL	0.144**	2.572	H1b supported
SPI → ETL	0.501***	8.025	H2a supported
SPI → ERL	0.554***	8.564	H2b supported
ETL → CAP	0.245***	3.839	H3a supported
ERL → CAP	0.235**	3.347	H3b supported
ETL → AAP	0.255**	3.902	H4a supported
ERL → AAP	0.203**	2.922	H4b supported
CAP → PI	0.582***	12.236	H5a supported
AAP → PI	0.228***	3.725	H5b supported

***Significant at the 0.001 level; **significant at the 0.01 level.

*SPO, social presence of others; SPI, social presence of interactions; ETL, exploitative learning; ERL, exploratory learning; CAP, cognitive appraisal; AAP, affective appraisal; PI, purchase intention.*

The study hypotheses imply several mediation paths, with social learning external and internal process mediating the relationship between social presence and PI. We further analyzed whether social learning process played an intermediary role with Bootstrap method (Lau and Cheung, 2012). The chain mediation effect is significant if 95% confidence interval does not include zero (Preacher and Hayes, 2008). The results suggested that zero was not included in 95% confidence interval for all mediations, showing the existence of mediation influence (see Table 5).

**TABLE 5:** Results for mediation testing.

Mediation paths	95% confidence interval	Path coefficient
SPO → ETL → CAP → PI	[0.012, 0.060]	0.030
SPO → ERL → CAP → PI	[0.007, 0.044]	0.020
SPO → ETL → AAP → PI	[0.004, 0.026]	0.012
SPO → ERL → AAP → PI	[0.002, 0.020]	0.007
SPI → ETL → CAP → PI	[0.029, 0.122]	0.071
SPI → ERL → CAP → PI	[0.034, 0.146]	0.076
SPI → ETL → AAP → PI	[0.013, 0.053]	0.029
SPI → ERL → AAP → PI	[0.008, 0.060]	0.026

*SPO, social presence of others; SPI, social presence of interactions; ETL, exploitative learning; ERL, exploratory learning; CAP, cognitive appraisal; AAP, affective appraisal; PI, purchase intention.*

## Discussion

### Discussion of the Empirical Results

The objective of this study was to explore the influence mechanism of social presence on PI in brand live streaming commerce, to help brand marketers and sellers to achieve sustainability in live streaming marketing. We sought to this objective by integrating social cognition theory, social presence theory, and social learning theory. Through collecting data from qualified participants, the research showed that social presence was significantly related to social learning process which promoted PI. Our study represents several findings as follows.

First, both SPO and SPI positively influence ETL and ERL. Social presence is favorable to establish mutual trust between users in medium (Ou et al., 2014). The social cues such as real-time comments, consultations, and also their “purchasing” and “like” behaviors in live streaming space make consumers’ perception of the existence of others. Additionally, live chat tool conveys a sense of interactions. Both of the perception of others’ presence and interactions in social commerce environments can increase consumers’ trust on sellers (Lu et al., 2016) and then affect their engagement behaviors such as interpersonal communications (Wongkitrungrueng and Assarut, 2020). Consumers can understand the ability and integrity of the seller according to others’ information and through interactions, which is further conductive for consumers to learn more information from the seller. Specifically, consumers may have questions or doubts on products experienced by streamer, or they may prefer to learn new information about other products or other aspects. The higher degree of others’ presence or interactions is, the more probably for consumers to interact with sellers to learn information they need. Therefore, SPO and SPI in live streaming commerce can affect interpersonal interactions between consumers and sellers, thus generating more external learning process of ETL and ERL.

Second, both ETL and ERL have significant influences on CAP and AAP. This is consistent with previous studies which suggested that members engage in social environment mainly because the content of social networks can provide informational support and emotional support (Chen et al., 2013). The utilitarian information that conveyed in live streaming commerce will contribute to reduce uncertainty in consumers’ decision-making process. Besides, Habibi et al. (2014) point out that consumers can feel themselves as part of the brand through interactions and directly promote positive emotions toward the brand. Social environment is favorable for the information sharing and exchanging (Ashley and Tuten, 2015). In live streaming commerce, consumers can gain the experiential information of streamer and other customers and also explore more new information that meets their own needs and preferences by deeply consulting or making comments. Such process can positively influence consumers’ psychological process of cognitive and affective evaluations toward sellers.

Third, both CAP and AAP have impacts on consumers’ PI, which is in line with prior studies (Chen A. H. et al., 2017). Consumers always make their purchase decision based on both knowledge structures and feelings (Lee et al., 2012). Zhao et al. (2019) also demonstrate that the positive attitude toward the seller and brand also significantly affects consumers’ PI.

### Theoretical Contributions

This study contributes to research in several ways. First, the study increases understanding on consumers’ purchase decision in live streaming commerce context from social presence perspective, although previous studies have investigated the factors affecting customers’ PI in live streaming commerce from perspectives of IT affordance, atmosphere clues, interact celebrity characteristics, etc. (Sun et al., 2019; Xu et al., 2019; Li et al., 2020). In addition, Ang et al. (2018) also show that live streaming is more beneficial to generate social presence than prerecorded video and further affects information search and subscription intention toward the platform. However, compared with traditional e-commerce, the interaction in live streaming commerce is real time and multidirectional, and these characteristics have not been adequately reflected in previous social commerce research. Thus, from this perspective, this study enriches and deepens the application of social presence theory in new marketing environment.

Second, this paper introduces social learning theory and perspective in the field of organizational learning into marketing research domain, extending the application scope of social learning theory. Most of the studies on the influence mechanism of social presence on PI were from the perspectives of trust, perceived value, experience, perceived uncertainty, and perceived usefulness (Ang et al., 2018; Osei-Frimpong and Mclean, 2018). Meanwhile, the relationship between social presence and social learning is mostly discussed in e-learning research (Tu, 2000), with rarely explored in online marketing environments. This study empirically tests the roles of social learning process (external interaction process and internal psychological process) in the relationship between social presence and consumers’ behavior intention.

Third, this study focuses on CVL conceptualized by Myers (2018) and considers two dimensions of CVL according to ambidextrous learning (Gibson and Biekinshaw, 2004). Live streaming commerce provides a new way for consumers to obtain information, and social learning process will occur by both observing and interacting (Li et al., 2020). Most previous research examined observational learning, such as learning from reviews and comments, recommendations and referrals, others’ purchase behaviors, and so on. Additionally, these studies basically emphasize the impact of independent learning on consumers’ passive behavior like PI, without deeply exploring its influence on consumers’ active interactions with sellers. This study makes up this research gap to some extent with focusing on the effects of perception of others on consumers’ active interactions, especially their CVL. Moreover, since perceived interactivity has a positive effect on parasocial interaction (Labrecque, 2014), the influence of perception of interaction with sellers on customers’ CVL is also examined. Additionally, this study proves the positive effect of CVL as an external learning process on consumers’ internal psychological process and PI as well.

### Practical Implications

This study also has practical significance. First, it is necessary for brand marketers to motivate consumers’ PI by attaching importance to factors that stimulate their cognitive and affective status. This paper suggests that both CAP and AAP positively affect consumers’ PI. Although many sellers have tried to stimulate consumers’ perceived emotional value through promotions or cooperating with internet celebrities in live streaming marketing strategies, they also need to pay enough attention to customers’ utilitarian value after attracting them. That is, useful content and interactions need to be taken into account for brands to make consumers to obtain necessary knowledge and better emotional experience in live streaming.

Second, the results in this paper show the social learning process mechanism which provides brand marketers a practical guidance. For online sellers, it can stimulate consumers’ AAP and maintain their loyalty through communicating with consumers and making them interact with other members (Chen A. et al., 2017). Chen and Lin (2018) also point out that interactions have significant impact on positive attitude toward the seller. Marketers can motivate consumers to learn product knowledge through favorable interactions, so as to stimulate consumers’ positive CAP and AAP, and promote their PI, which is conducive to the sustainable development of brand live streaming marketing.

Third, the technological characteristics in live streaming commerce provide consumers with more opportunities and conditions for vicarious learning (Sun et al., 2019). Both SPO and SPI have significant influences on consumers’ CVL. On the one hand, it is necessary to attach importance for brands to the existence and interactions of consumers such as “likes,” product evaluations and consultations, and purchase behavior. For instance, sellers could carry out various kinds of interactive activities or encourage consumers to participate in topics linked with products to increase SPO. Furthermore, perception of others may foster trust toward the seller and originate herd behavior like learning brand knowledge through active interactions with the seller where CVL would happen. On the other hand, the interactivity conveyed by the seller is also critical, since the higher degree of perception of interactions with seller, the more tendency for consumers to talk about products and their demands more deeply and comprehensively. For example, besides the streamer, an additional professional may be necessary to reply consumers’ questions and consultations timely and effectively.

### Limitations and Future Research

This paper has several limitations. First, it may limit the generality of the findings since this study only considered consumers who had experience of watching brand live streaming in Taobao which only stands for one category of live streaming commerce (i.e., adding live streaming tools in e-commerce website). Future studies could be conducted focusing on other types of live streaming commerce platforms (e.g., Instagram, Facebook, Twitter, Douyin) to extend current research scope. Second, there is no distinction between product types in this study. For various product categories (e.g., hedonic and utilitarian items, search and experience products, and goods and services), the relationships among social presence, social learning process, and PI may be different. Third, social presence can be regarded as a media attribute conveyed by social technologies, and Zhang et al. (2014) confirmed that technological features in social commerce have positive impact on social presence. Based on the various antecedents that impact social presence proposed by Oh et al. (2018), future research could explore the influences of other factors on different dimensions of social presence in live streaming commerce. Finally, Myers (2018) divided vicarious learning into two categories of independent vicarious learning and CVL, and this research only focused on the latter type. Independent learning from different sources in social commerce can affect users’ psychological process and PI (Chen A. H. et al., 2017). Since observing comments and social behaviors delivered by others can be regarded as independent vicarious learning, it would be very interesting to investigate and compare the two different types of vicarious learning in live streaming commerce, namely accepting information independently and passively and also obtaining information by interacting actively.

## Data Availability Statement

The original contributions presented in the study are included in the article/supplementary material, further inquiries can be directed to the corresponding author/s.

## Author Contributions

ML and YH designed the research, wrote the manuscript, conducted the literature review, and built the conceptual model. ML analyzed the data. YH wrote the discussion and conclusion. Both authors contributed to the article and approved the submitted version.

## Conflict of Interest

The authors declare that the research was conducted in the absence of any commercial or financial relationships that could be construed as a potential conflict of interest.

## Publisher’s Note

All claims expressed in this article are solely those of the authors and do not necessarily represent those of their affiliated organizations, or those of the publisher, the editors and the reviewers. Any product that may be evaluated in this article, or claim that may be made by its manufacturer, is not guaranteed or endorsed by the publisher.

## Appendix

**TABLE A1:** Measurement scales.

Measurement scales
Social presence of others SPO1. There are many others feel interested with the products in live streaming. SPO2. There are many others sharing product-related information in live streaming. SOP3. There are many others who “are buying” the products in live streaming. Social presence of interactions SPI1. I can make sense of the attitude of sellers by interacting *via* live streaming. SPI2. I can imagine what are they like by interacting *via* live streaming. SPI3. There is a sense of human touch to communicate with sellers *via* live streaming. SPI4. Communication *via* live streaming was warm. Exploitative learning ETL1. Upgraded current knowledge for products introduced. ETL2. Upgraded experience knowledge for products introduced. ETL3. Enhanced abilities in solving problems occurrence in product application. ETL4. Strengthened the knowledge and skills to improve the efficiency of purchase decision. Exploratory learning ERL1. Acquired basic knowledge of new products. ERL2. Acquired experience knowledge of new products. ERL3. Learned totally new skills in using skills of products. ERL4. Strengthened skills in areas where it has no prior experience. Cognitive appraisal CAP1. The live streaming conducted by the seller was effective for achieving the goal of your visit. CAP2. The live streaming conducted by the seller was convenient for attaining the goal of your visit. CAP3. You felt comfortable *via* live streaming conducted by the seller to achieve the goal of your visit. CAP4. The live streaming conducted by the seller was helpful for achieving the goal of your visit. Affective appraisal Your overall experience with the live streaming conducted by the seller was as follows: AAP1. Happy AAP2. Good AAP3. Relaxed AAP4. Likable AAP5. Satisfactory Purchase intention PI1. I would purchase products from the seller in the future. PI2. The seller is my first choice to buy relative products. PI3. I would do more businesses with this seller in the next few years.
